# Supplementary material for: Simple autonomous agents can enhance creative semantic discovery by human groups
Source: Nat Commun. 2024 Jun 18;15:5212. doi: 10.1038/s41467-024-49528-y (PMC11189566; doi:10.1038/s41467-024-49528-y)
Supplement: Supplementary file 1 — Supplementary Information [file 41467_2024_49528_MOESM1_ESM.pdf]

**Supplementary material for**
**"Simple Autonomous Agents Can Enhance Creative Semantic Discovery by Human Groups"**

## Supplementary Methods

### Collection of the 20,000 nouns

The specific way we collected the 20,000 nouns was as follows:

1. Select a word from word2vec[1,2] based on its frequency of occurrence (how often it appears) in the corpus.
2. Use NLTK[3] and wordnet[4] to check if the obtained word is a singular noun and does not contain "_," "www," or "@."
3. If the word passes these filters and is not a swear word (see below), it is added to the list.
4. Repeat this procedure until the list contains 20,000 words.

We excluded certain words when constructing the 20,000-word list in order to avoid any potential distraction to the participants during the game. More specifically, we did not include the following words even though these words were frequent in the corpus: "ass", "bitch", "cum", "foursome", "fuck", "molestation", "shit", "rape", "rapes", "rapist", "threesome", "bullshit", "asshole", "retard", "xxx", "chink", "fagot", "nigger", "nigga", "negro", "fucker", "dumbass", "motherfucker", "shite", "cunt", "macaca", "coon", "fucker", "arsehole", "spick", "gook", "horseshit", and "squaw."

### Selection of 18 target nouns

To select target nouns, we used a clustering method[5] to divide the 20,000 nouns into 25 semantically distinct clusters using the vector representation of each noun from word2vec. Using data from pilot experiments, we then removed 5 clusters from our further consideration because they contained nouns that were similar to participants' typical initial answers (e.g., animal names such as "dog" and "cat"), and we expected that the game would be too easy if we chose target nouns from these clusters. Next, we chose a noun from each of the remaining 20 clusters, ensuring that the frequency of these 20 nouns in the whole corpus was similar (the frequency of the target nouns in the corpus all fit within a 5% window). Finally, we removed two nouns ("plateaus" and "nu") from the list of 20 chosen nouns because "plateaus" was a plural noun (we used NLTK to remove plural nouns, but some plural nouns still passed the filter) and "nu" came from a cluster made up of nonsense words.

In the end, we were left with 18 target nouns that (1) are semantically distant from each other; (2) are semantically different from the typical initial selections of human

participants; and (3) have similar frequency in the corpus. The following 18 target nouns were used in the experiment: “recce”, “cartography”, “investiture”, “comedown”, “hesitance”, “decile”, “shoehorn”, “edutainment”, “narrowness”, “activewear”, “epee”, “doyenne”, “actuation”, “sarcoma”, “braggadocio”, “jowl”, “fratricide”, and “translocation.”

**Details regarding participant recruitment**

Following our pre-registered plan, we recruited 125 groups, each consisting of 15 participants (1,875 participants). This resulted in 25 unique groups for each of the five landscape variables, including the no-decoy, short/narrow, short/wide, tall/narrow, and tall/wide landscapes.

After the initial data analysis, we found that the 25 groups assigned to the tall/wide landscape performed exceptionally well overall (Supplementary Fig. 8). We conjectured that this was statistical noise based on our common-sense prediction that the tall/wide landscape should have been the most challenging landscape. To confirm this conjecture, we decided to deviate from the pre-registered plan and recruit another 25 groups (375 participants) only for the tall/wide landscape while following the same methods as in the recruitment of the original 25 groups. Supplementary Fig. 8 shows the results from the additional 25 groups for the tall/wide landscape; we used the additional 25 groups assigned to the tall/wide landscape for the main analyses, combined with the data for the other four landscapes, resulting in 125 unique groups consisting of 1,875 participants. However, a meta-analytic approach that used all 50 groups in this category did not yield materially different overall results. That is, we replicated the main analyses using both the original and additional groups that were assigned to the tall/wide landscape and found no significant differences. This can be seen in Supplementary Fig. 8.

**Participants demographic information**

After completing the main task, participants were asked to provide their demographic information, including their gender and age. Out of the 1,821 participants who completed the post-session questionnaire, 953 identified as male, 848 as female, 12 as non-binary, and 8 preferred not to answer. The mean age of participants was 36.04 (s.d. = 11.22).

**Probability of impact of decoy landscapes**

Before running the experiment, it was unclear if the height of a local maximum would be more or less impactful than the width. The results strongly indicate that the width is far more important than the height, but in order to make that claim, we wanted to have two landscapes with different heights and widths that had roughly the same level of manipulation. To this end, we developed a method to approximate the probability of impact, meaning the probability that an individual would be led astray or distracted by the decoy peak in the early stages of the game.

To compute this probability, we consider an individual with three neighbors, the average in our networks. Next, we assume these players all choose nouns randomly for 10 rounds. If, at any point in those 10 rounds, the focal individual's highest point value word seen so far is a boosted word, we say that the peak has impacted the focal individual.

We estimated this probability of impact using simulations. For each target/decoy pair, we ran 100 simulations, and concluded that when $\mathrm{boost}_{\mathrm{num}} = 6{,}000$ and $\mathrm{boost}_{\mathrm{coef}} = 0.975$ (tall/narrow) or when $\mathrm{boost}_{\mathrm{num}} = 12{,}000$ and $\mathrm{boost}_{\mathrm{coef}} = 0.85$ (short/wide), the probability was approximately 66%.

**Pairings of target and decoy nouns**

After selecting the 18 target nouns, we wanted every target to have a unique decoy noun to create rugged landscapes. Ideally, these words would be far apart in the semantic space so that the decoy noun would be boosted very far up the ranking. The 18 targets and 18 decoys formed a bipartite graph, and maximizing the distances between them required solving a maximum weight matching for a bipartite graph.

For each of the 18*17 potential target/decoy pairs, the weight of that edge in the bipartite graph is the rank (out of 20,000) of the decoy with respect to the target. Many algorithms have been developed to solve such computational problems[6], and the python scipy package[7] we implemented used the Jonker-Volgenant algorithm. The resulting Supplementary Table 1 shows the set of target/decoy pairs that maximizes the total rank of all the decoys with respect to their targets. As a note, the weight matrix is not symmetric and therefore these target/decoy pairs are not symmetric, e.g. braggadocio's decoy is translocation, but translocation's decoy is doyenne.

**Boosting of decoy nouns**

When designing our boosted landscapes, we wanted our algorithm to have a few desirable properties. First, we wanted our algorithm to have two parameters: decoy peak width and decoy peak height. The width represented the number of words boosted, and the height represented how far up the rank the decoy moved. Second, we wanted the nouns near the decoy to be boosted farther than those far away from the decoy. This led to our third concern, which was that we needed to ensure that after boosting all the nouns close to the decoy, the target was still the highest valued noun. Because of the high dimensionality of the space, even if the decoy was very far from the target, there were often words very close to both the target and the decoy. These words needed to move far up the ranks because they are close to the decoy, but this tended to boost them past the target, which was unacceptable. Finally, we wanted our algorithm to be agnostic to the order in which the words were boosted.

Next, we describe the algorithm and demonstrate it on a small example with 20 words,

three of which are boosted. Let $\mathrm{boost_{num}}$ be the number of words that are being boosted. The height of the decoy peak is determined by $\mathrm{boost_{coef}}$, a number between 0 and 1 which represents how close to the target the decoy is boosted. Finally, $\mathrm{boost_{pos}}$ specifies the order in which we boost our words and is determined by distance to the decoy, with the decoy which has $\mathrm{boost_{pos}} = \mathrm{boost_{num}}$ all the way to the boosted word farthest from the decoy with $boost_{pos} = 1$. Of course, each word also has an $\mathrm{rank_{old}}$, its rank prior to boosting. An intermediate step, placements, is used where boosted words move up, before all ties are broken and the final $\mathrm{rank_{new}}$ is determined for each word.

The algorithm begins with the target and moves its way down the rank. For each word, if the word is unboosted, its placement is simply $\mathrm{rank_{old}}$. If the word is boosted, then $\mathrm{placement} = \left\lceil \mathrm{rank_{old}} + \left(\frac{\mathrm{boost_{pos}}}{\mathrm{boost_{num}}}\right) \mathrm{boost_{coef}}(\mathrm{n} - \mathrm{rank_{old}}) \right\rceil$. Many words may have the same placements. To break these ties, if two words have the same placement, the word with the higher $\mathrm{rank_{old}}$ also has the higher $\mathrm{rank_{new}}$.

To demonstrate this algorithm, suppose we have the example in Supplementary Figure 2 with 20 words, five of which are shown in Supplementary Figure 2a. (The full rank of all 20 words is shown in Supplementary Figure 2b.) Word A is very far from the decoy and therefore does not get boosted at all. Words B and C, together with the decoy, are being boosted. Word B is closer to the decoy, so it should be boosted more than Word C. However, it is also close to the target, which will reduce the number of ranks it actually moves up, because boosting is inversely proportional to $\mathrm{rank_{old}}$. Suppose $\mathrm{boost_{num}} = 3$ and $\mathrm{boost_{coef}} = 0.6$.

For Decoy, $\mathrm{placement} = \left\lceil 9 + \left(\frac{3}{3}\right) 0.6(20 - 9) \right\rceil = \lceil 9 + 6.6 \rceil = 16.$

For Word B, $\mathrm{placement} = \left\lceil 16 + \left(\frac{2}{3}\right) 0.6(20 - 16) \right\rceil = \lceil 16 + 1.6 \rceil = 18.$

For Word C, $\mathrm{placement} = \left\lceil 4 + \left(\frac{1}{3}\right) 0.6(20 - 4) \right\rceil = \lceil 4 + 3.2 \rceil = 8.$

For all other words, $\mathrm{placement} = \mathrm{rank_{old}}$. The placements are shown in the middle column of Supplementary Figure 2b. Then, for each placement with multiple words, $\mathrm{rank_{new}}$ is determined by ordering all words in that placement according to $\mathrm{rank_{old}}$. Notice that Word B only moved up one rank despite being closer to decoy than Word C which moved up three ranks, because it was already very close to the target.

Supplementary Fig. 2 c and d provide 3D and 2D illustrations of the landscapes, respectively. Supplementary Table 1 describes the boosted rank of decoy nouns in each decoy landscape.

**Fractional factorial design**

Each group played five games with five different bot treatments. The order of the five bot treatments was delivered based on a fractional factorial design. There were five bot

treatments (least similar, most similar, random, no bot, and solo) and we varied the order in which these five treatments appear. There are 5!=120 different possible orders. In this design, each treatment appears first (second/third/fourth/fifth) in one fifth of the orders.

This is a very desirable property to avoid order effects, but we did not want to do 120 experiments for each decoy landscape, so instead we created blocks of five orders such that, within each block, each treatment appears first (second/third/fourth/fifth) exactly one time. There are $120 * 199 * 198 * 197 * 196 = 182{,}566{,}802{,}880$ possible ordered lists of five orders, but only 161,280 of them have this property. For ease of reading, we replaced the five bot treatments (least similar, most similar, random, no bot, and solo) with the numbers 0 through 4, respectively. We can represent the block as a five-by-five matrix where each row is an order, in which case our block has the desired positional property if each number appears exactly once in each row and column. These configurations, known as Latin Squares, are well-studied. The first block is below:

$$A = \begin{matrix} 3 & 1 & 4 & 2 & 0 \\ 4 & 2 & 3 & 0 & 1 \\ 2 & 0 & 1 & 4 & 3 \\ 0 & 3 & 2 & 1 & 4 \\ 1 & 4 & 0 & 3 & 2 \end{matrix}$$

Ultimately, we wanted to choose five of these blocks (for a total of 25 orders), but if we just randomly sample five blocks, it is likely that the same order will appear in multiple blocks. We avoided this by sampling blocks in sequence, discarding a potential block if it contained an order that already appeared in a previous block. These are the remaining four blocks, each sampled one at a time:

$$B = \begin{matrix} 4 & 0 & 3 & 2 & 1 \\ 0 & 2 & 4 & 1 & 3 \\ 1 & 4 & 2 & 3 & 0 \\ 3 & 1 & 0 & 4 & 2 \\ 2 & 3 & 1 & 0 & 4 \end{matrix}$$

$$C = \begin{matrix} 3 & 2 & 1 & 0 & 4 \\ 4 & 1 & 0 & 3 & 2 \\ 1 & 4 & 3 & 2 & 0 \\ 2 & 0 & 4 & 1 & 3 \\ 0 & 3 & 2 & 4 & 1 \end{matrix}$$

$$D = \begin{matrix} 1 & 4 & 0 & 2 & 3 \\ 2 & 3 & 4 & 0 & 1 \\ 4 & 0 & 3 & 1 & 2 \\ 3 & 1 & 2 & 4 & 0 \\ 0 & 2 & 1 & 3 & 4 \end{matrix}$$

$$
E = \begin{matrix} 0 & 4 & 3 & 2 & 1 \\ 1 & 3 & 0 & 4 & 2 \\ 4 & 2 & 1 & 3 & 0 \\ 2 & 1 & 4 & 0 & 3 \\ 3 & 0 & 2 & 1 & 4 \end{matrix}
$$

The final result is a random set of five blocks with no orders in common. We used another Latin Square to determine the order in which each decoy treatment went through the five blocks.

| Experiment #: | 1 – 25 | 26 – 50 | 51 – 75 | 76 – 100 | 101 – 125 |
|---|---|---|---|---|---|
| tall and wide: | A | E | D | C | B |
| tall and narrow: | B | C | E | D | A |
| short and wide: | D | A | B | E | C |
| short and narrow: | C | D | A | B | E |
| no decoy: | E | B | C | A | D |

By giving each decoy landscape a different order in which to go through the blocks, we ensured that, after the first 25 (50/75/100/125) experiments, each block has been used once (twice/three times/four times/five times). From this, it follows that, after 25 (50/75/100/125) experiments, each of the 25 orders has been used once (twice/three times/four times/five times). Within each block, we also randomized the order in which each treatment uses the five orders. Finally, every five experiments, each decoy landscape was done once. However, we randomized the order of decoy treatments for each set of five to avoid any order effects while still ensuring that after the first 5 (10/15/20/…) experiments, each decoy landscape has been used the same number of times. For the actual orders of the experiment, see the preregistration.

We confirmed that the main results presented in Fig. 2 were qualitatively unchanged when the order of the most similar bot condition (ranging from game 1 to game 5) was included as a continuous fixed effect in the regression (Supplementary Fig. 9).

**Consideration of the influence of typos**

In the experiment, submitted answers were only accepted as valid nouns when they were included in the list of 20,000 nouns. However, nouns classified as invalid could just be a typo. Accordingly, we tested the frequency of typos in the experiment by randomly sampling 200 invalid answers from the entire experiment, checking for typos using the R package hunspell v. 3.0.2, and examining them manually. Results indicated that 30 out of the 200 invalid nouns (15%) could have been typos. Because invalid answers accounted for 7.5% of all the submitted answers in the experiment, typos would be estimated to only account for 1.1% (15% * 7.5%) of all the submitted answers. Thus, we would not believe that typos had a substantial effect to the extent that it might change the main conclusion.

## Supplementary Figures

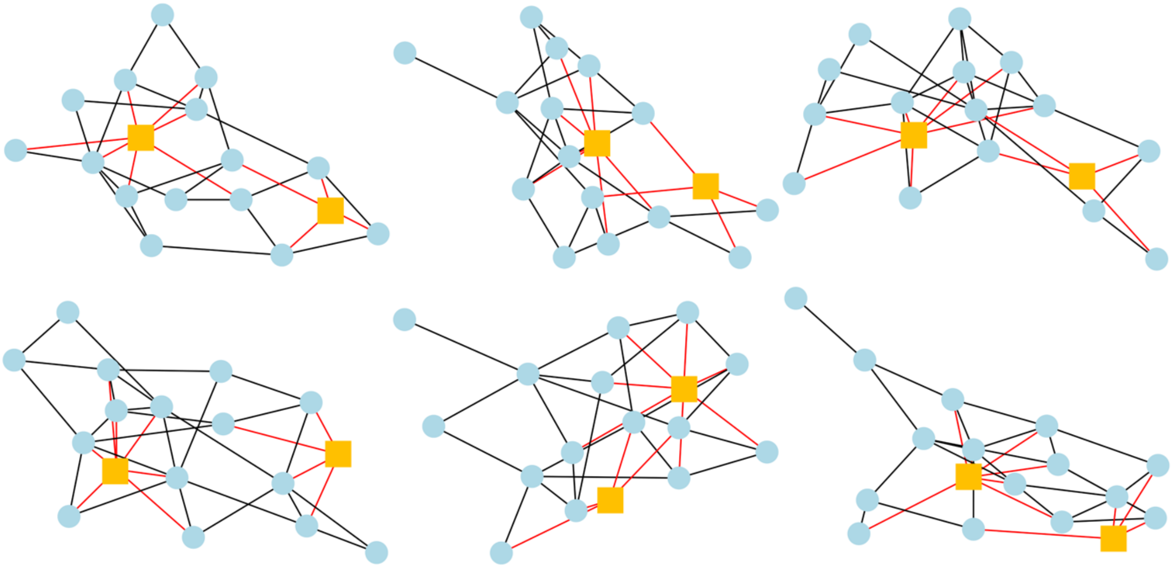

Supplementary Fig. 1 | The six social networks used in the experiment. Each network consisted of 15 human players (light blue nodes) and two bots (yellow squares). The two bots had 7 and 4 connections, respectively, by design. The connections of each bot are highlighted by red ties which were removed in the no-bot condition. In the solo condition, all the edges in the network were removed. One of these networks was randomly assigned to each experimental group. The main results were qualitatively unchanged when network selection was included as a fixed effect in the regressions.

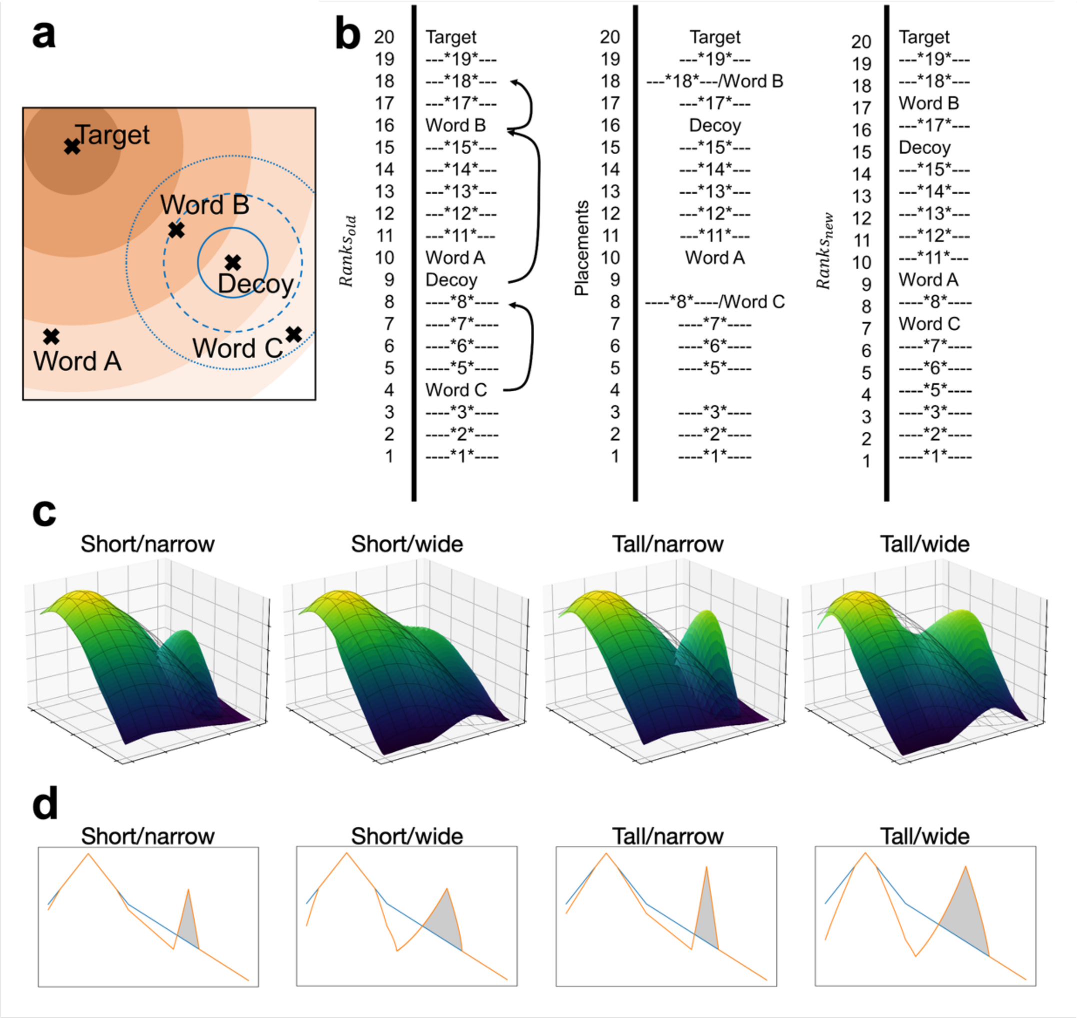


Supplementary Fig. 2 | **a**, Illustrative 2D example of a word space. A decoy and two nearby words, Word B and Word C, are being boosted. Word A is too far away from the decoy to be boosted. **b**, An example of the boosting algorithm applied to the landscape in (a). First, the decoy and words B and C move up in the placements proportional to their distance from the target and closeness to the decoy. After placements are determined, the new ranking is found by breaking all ties where words have the same placement. **c**, 3-dimensional examples of the four decoy landscapes. Regardless of the landscape, the rank sum was unchanged. The black mesh shows the ranks prior to boosting. Each landscape in this illustration was formed by creating a 100x100 grid in the unit square, placing the target at (0.2, 0.2), the decoy at (0.7, 0.7), and then using Euclidean distance to rank all points on the grid with respect to the target, breaking ties randomly. **d**, 2-dimensional examples of the decoy landscapes. This time, we used 10,000 points uniformly spaced on the unit interval and used Euclidean distance to rank them, with ties broken randomly. In these examples, the probability that the decoy had an impact can be approximated as the grey shaded area. The experimental parameters were chosen so that the tall/narrow landscape had the same total probability of impact as the short/wide landscape. In (c) and (d), the vertical axis represents the rank of a word after boosting. The boost numbers were 1,500 and 5,000 and the boost coefficients were 0.6 and 0.85. Words that are far from the target and the decoy may not be boosted at all. And if there are no words below them that are close to the decoy that can pass them after boosting, their ranks may be unchanged. This happens in the bottom right corner of our examples here, but happens rarely in the high-dimensional noun semantic space.

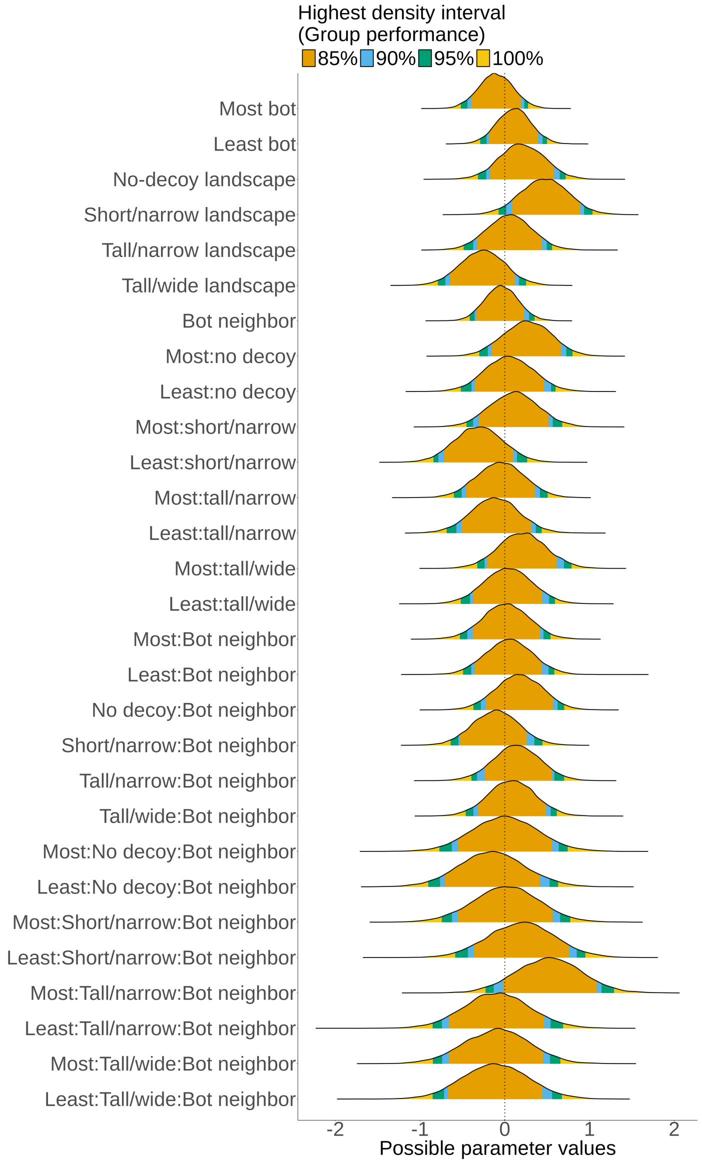


Supplementary Fig. 3 | Posterior distributions of regression coefficients with computed HDIs using the averaged cosine similarity between answers and the target for each game as the dependent variable. Unlike the results presented in Fig. 3b in the main analysis, this analysis included the 'Bot neighbor' variable, which indicates whether individuals were connected with a bot (coded as 1) or not (coded as 0), as a discrete fixed effect in the regression. The sample size is the same as in the results presented in Fig. 3b ($n$ = 625). The regression did not reveal statistically meaningful differences in the task performance with respect to this variable.

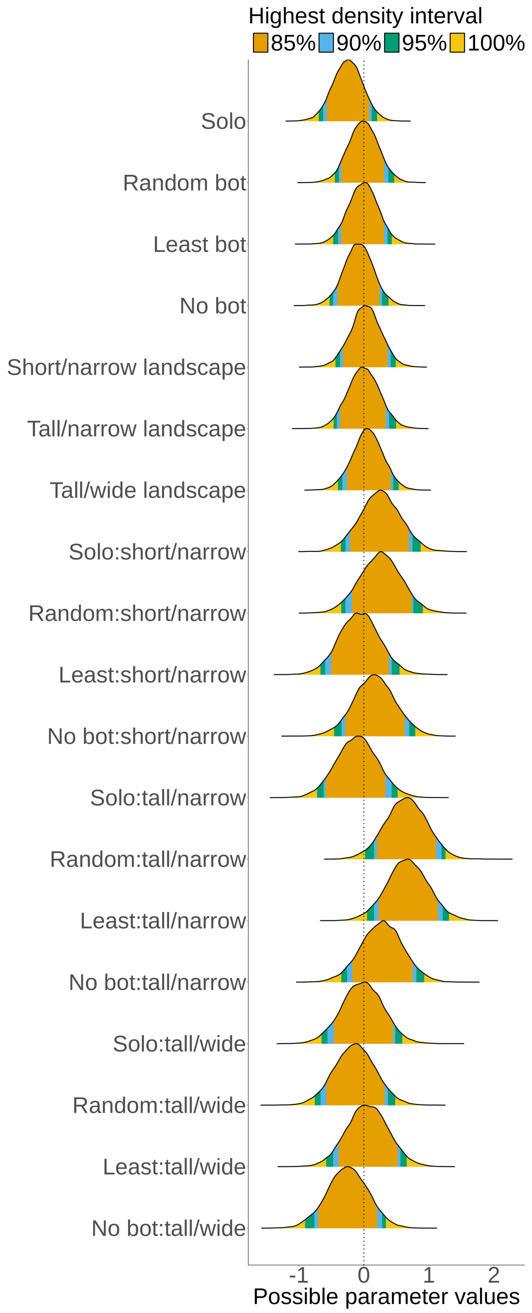


Supplementary Fig. 4 | Posterior distributions of regression coefficients with computed HDIs using the averaged cosine similarity between answers and the decoy for each game as the dependent variable. Out of 125 groups, the 25 groups assigned to the no-decoy landscape were necessarily excluded from this analysis, resulting in 100 unique groups that each completed 5 games. Thus, the number of data points was 500 in this analysis ($n = 500$). The regression model's independent variables included fixed effects of bot conditions, landscape variables, and their interactions, with the reference variables being the no-bot condition and short/wide landscapes.

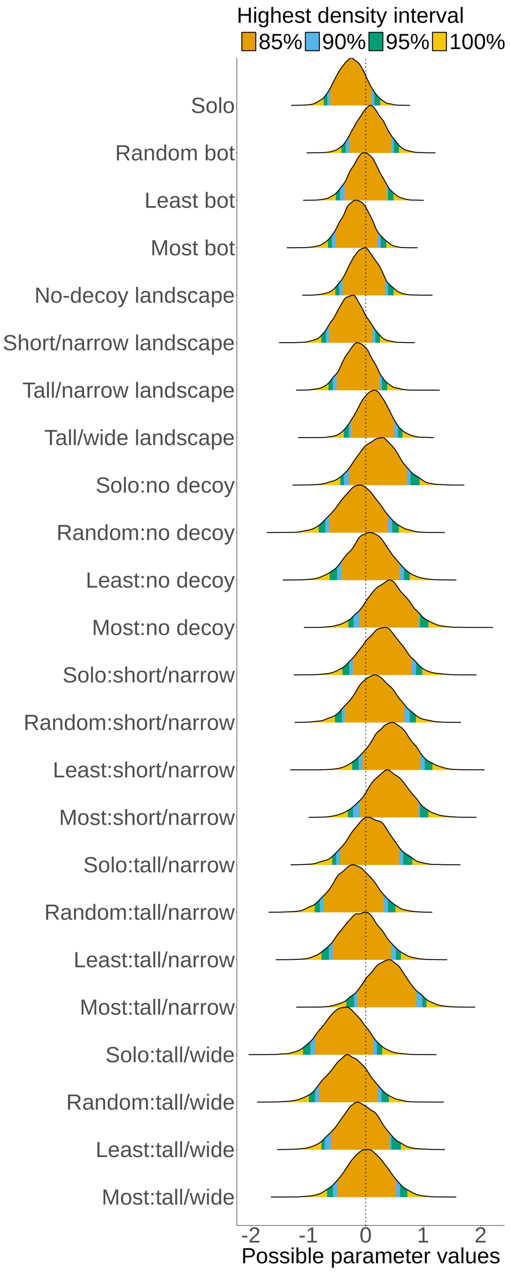


Supplementary Fig. 5 | Posterior distributions of regression coefficients with computed HDIs using the closest noun to the target over the course of the entire game as the dependent variable. The study incorporated 125 unique groups, each completing 5 games, resulting in 625 data points ($n$ = 625). The regression model's independent variables included fixed effects of bot conditions, landscape variables, and their interactions, with the reference variables being the no-bot condition and short/wide landscapes. These results indicate that this task was simple enough that all groups (regardless of treatment) had at least one guess that was very close to the target noun, so using the best guess instead of the average guess would not allow enough variability in our results to make any statistical claims about the effect of treatment on a group’s best guess.

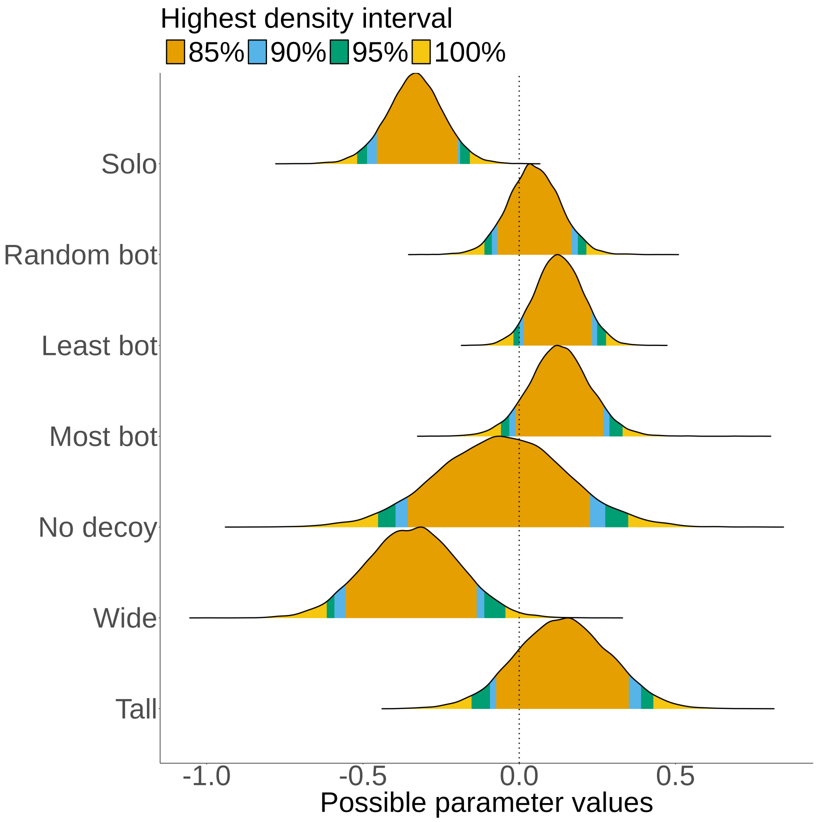


Supplementary Fig. 6 | Posterior distributions of regression coefficients with computed HDIs using the correlation between point values obtained in each round $t$ and the cosine similarity between nouns in rounds $t$ and $t$+1 as the dependent variable. Each of the 125 unique groups completed 5 games, resulting in 625 data points ($n = 625$).

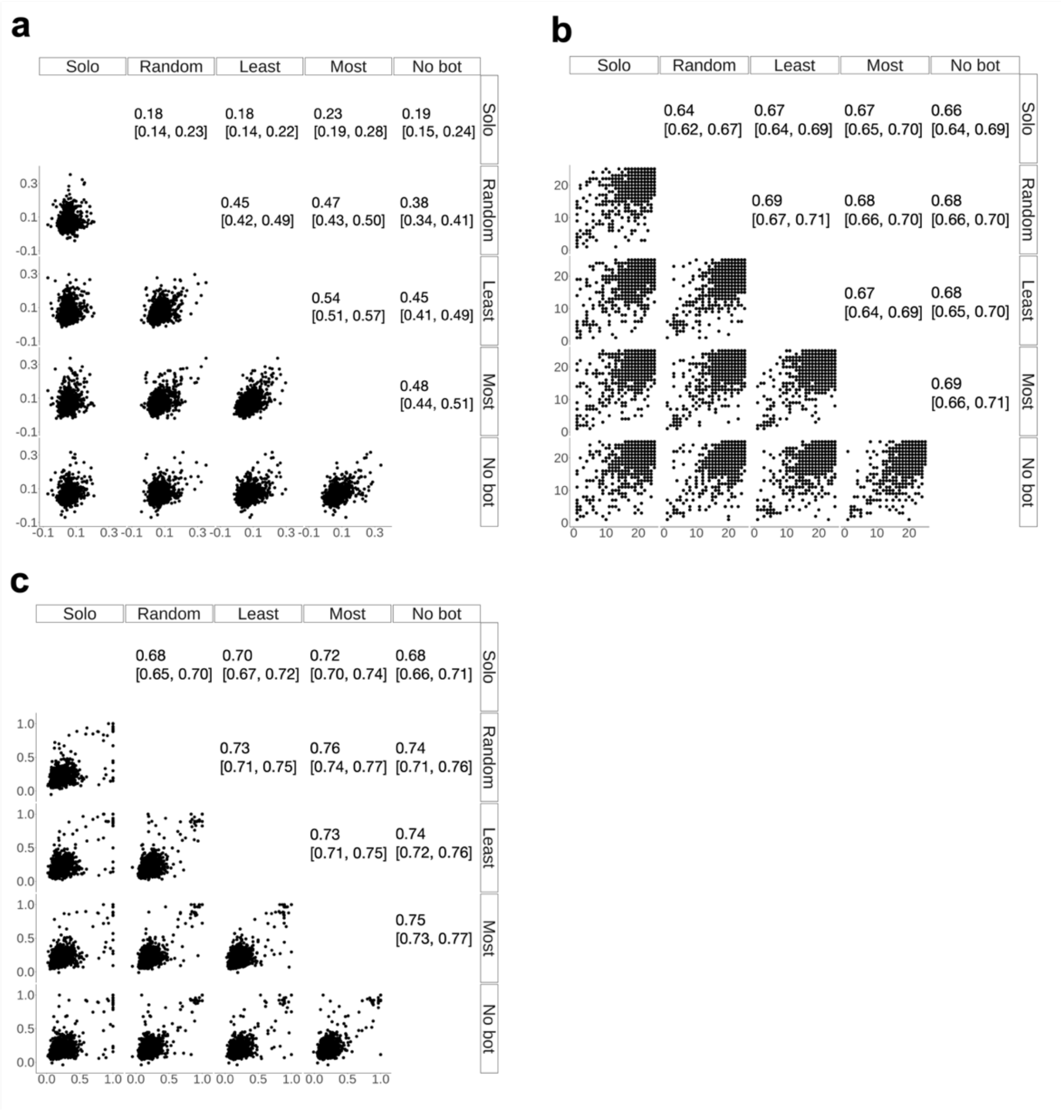


Supplementary Fig. 7 | **a**, Correlations between individual participant success across different bot treatments. For each participant and each game, we computed the average cosine similarity of all the participant's guesses with the target noun and then compared these values. For example, mean similarity with the target in the most-similar bot case is highly correlated with mean similarity with the target in the least-similar bot case, suggesting that successful players in one treatment were also successful in the other treatment. Participants who failed to yield correlation values in any of the five games were excluded, resulting in a total of 1,833 data points from 1,875 participants ($n$ = 1,833). **b**, Correlations between the number of unique nouns each participant answered in different games. The Pearson correlation coefficient was calculated for each pair along with the 95% highest density intervals presented in parentheses. The number of data point was 1,833 ($n$ = 1,833). Some data points are overlapping. **c**, Correlations between the extent of divergent thinking in each participant calculated by the cosine similarity between nouns answered in rounds $t$ and $t$+1 in different games. The number of data points was 1,812 ($n$ = 1,812). In all the panels, the Pearson correlation coefficient was calculated for each pair along with the 95% highest density intervals presented in parentheses.

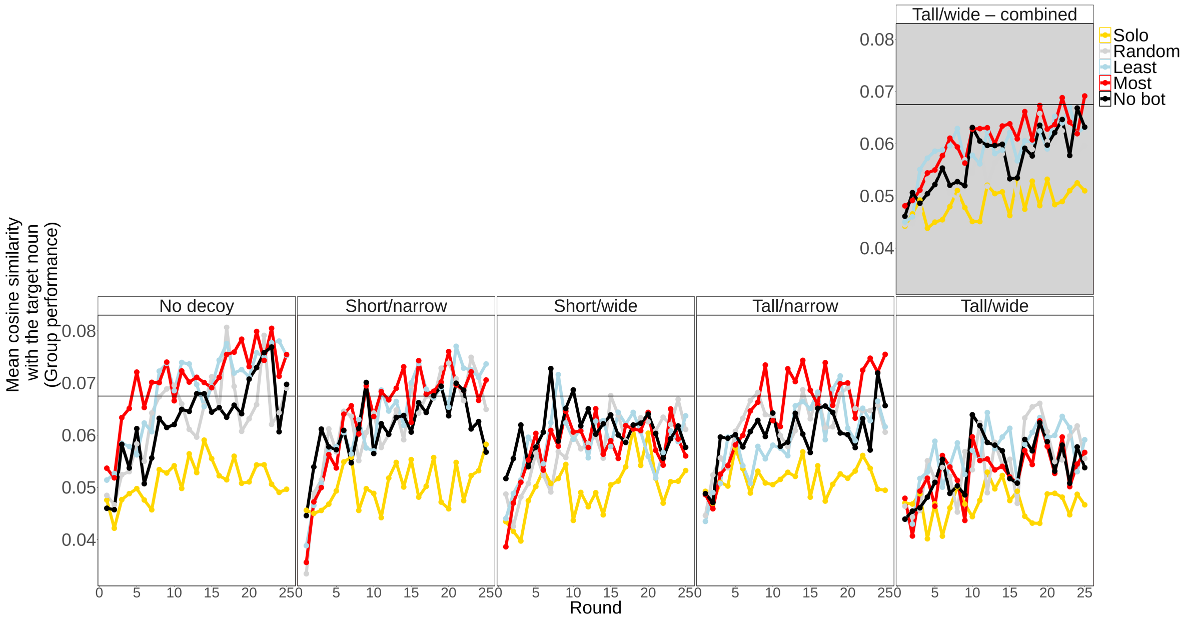


Supplementary Fig. 8 | Mean cosine similarity between participants' answer and the target noun across 25 rounds. The horizontal lines indicate the mean cosine similarity between each of the 18 target nouns and the 20,000 accepted nouns. This figure corresponds to Fig. 3a. The Tall/wide – combined facet, shown with gray background, shows the data that combined both the original and additional tall/wide landscape (i.e., $n$ = 50 groups).

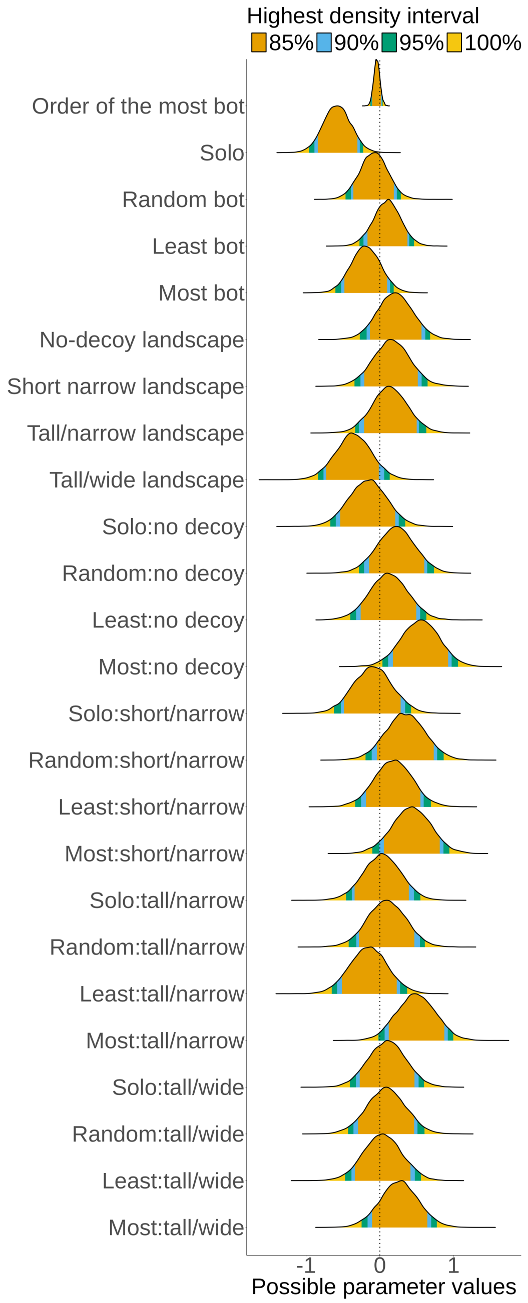


Supplementary Fig. 9 | Posterior distributions of regression coefficients with computed HDIs using the averaged cosine similarity between answers and the target for each game as the dependent variable. Unlike the results presented in Fig. 3b in the main analysis, this analysis included the order of the most-similar bot condition (ranging from game 1 to game 5) as a continuous fixed effect in the regression. This does not materially modify the results ($n = 625$).

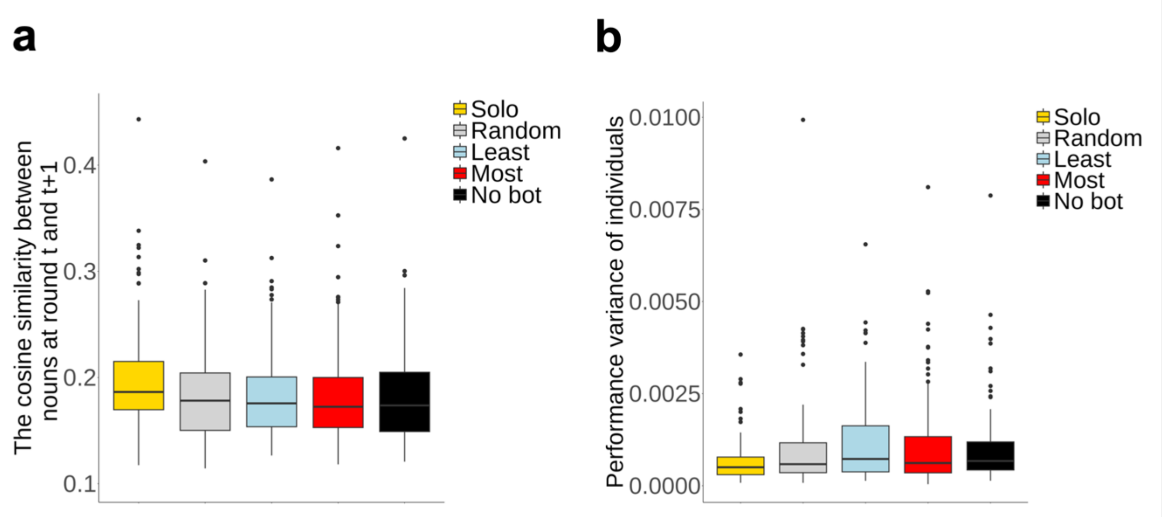


Supplementary Fig. 10 | **a**, The cosine similarity between nouns at round *t* and *t*+1 in the five bot conditions. The regression model's independent variables included fixed effects for the five bot conditions, with the reference variable being the solo condition ($n$ = 625). The results indicate that the cosine similarity between consecutive nouns was smaller in the solo condition than in the other conditions ($\beta_{Random}$ = -0.30, 95% HDI [-0.44, -0.16], $\beta_{Least}$ = -0.27, 95% HDI [-0.40, -0.13], $\beta_{Most}$ = -0.26, 95% HDI [-0.41, -0.09], $\beta_{No\ bot}$ = -0.31, 95% HDI [-0.45, -0.17]). **b**, The variance of the performance of 15 participants among the five conditions (most-similar bot, least-similar bot, random bot, no-bot, and solo condition). We first calculated the average performance of each participant in each of the five conditions across experimental groups. Then we calculated the variance of the performance for each group ($n$ = 625 data points of the performance variance). The regression model's independent variables included fixed effects for the five bot conditions, with the reference variable being the solo condition. The results indicate that the performance variance in each group was smaller when participants were in the solo condition (i.e., 15 isolated individuals) than when they were in other conditions ($\beta_{Random}$ = 0.39, 95% HDI [0.20, 0.58], $\beta_{Least}$ = 0.53, 95% HDI [0.35, 0.71], $\beta_{Most}$ = 0.47, 95% HDI [0.23, 0.73], $\beta_{No\ bot}$ = 0.41, 95% HDI [0.20, 0.61]). For the box-and-whisker plots, the box represents the interquartile range (IQR). The line within the box represents the median value. The upper (lower) whisker extends from the hinge to the largest (smallest) value no further than 1.5 * IQR from the hinge. Data outside the whiskers are plotted individually.

## Supplementary Tables

| Target | Decoy | Cosine similarity | Non-boosted decoy rank | Boosted decoy rank in SN | Boosted decoy rank in SW | Boosted decoy rank in TN | Boosted decoy rank in TW |
|---|---|---|---|---|---|---|---|
| activewear | fratricide | -0.02 | 2546 | 16664 | 15734 | 19422 | 19148 |
| actuation | shoehorn | -0.02 | 2968 | 16562 | 15635 | 19265 | 18927 |
| braggadocio | translocation | -0.01 | 2246 | 16339 | 15327 | 19188 | 18844 |
| cartography | comedown | -0.00 | 2687 | 16505 | 15547 | 19264 | 18928 |
| comedown | cartography | -0.00 | 1291 | 16321 | 15340 | 19285 | 18999 |
| decile | hesitance | -0.03 | 1447 | 16258 | 15305 | 19299 | 18993 |
| doyenne | recce | -0.08 | 2921 | 16465 | 15545 | 19284 | 18961 |
| edutainment | epee | -0.03 | 1869 | 16246 | 15221 | 19214 | 18810 |
| epee | edutainment | -0.03 | 7493 | 17344 | 16625 | 19486 | 19234 |
| fratricide | activewear | -0.02 | 2637 | 16460 | 15431 | 19379 | 19050 |
| hesitance | decile | -0.03 | 999 | 16115 | 15071 | 19166 | 18740 |
| investiture | jowl | -0.01 | 262 | 16060 | 14959 | 19247 | 18941 |
| jowl | investiture | -0.01 | 1970 | 16442 | 15455 | 19316 | 18954 |
| narrowness | sarcoma | 0.05 | 8133 | 17571 | 16850 | 19547 | 19300 |
| recce | braggadocio | -0.02 | 1264 | 16354 | 15324 | 19395 | 19171 |
| sarcoma | narrowness | 0.05 | 2580 | 16457 | 15477 | 19249 | 18880 |
| shoehorn | actuation | -0.02 | 1068 | 16196 | 15097 | 19385 | 19056 |
| translocation | doyenne | -0.05 | 1150 | 16507 | 15515 | 19422 | 19181 |

Supplementary Table 1: Pairing of target and decoy nouns. The average pairwise cosine similarity among the 18 target nouns is 0.067, which is less than the average between any two nouns out of the 20,000 nouns used in the experiment (0.078). Hence, the target nouns are relatively dispersed throughout the semantic space. Furthermore, we show that decoys typically have much lower cosine similarities with the targets than average and very low rank with respect to the targets. This confirms the success of our matching algorithm in creating very dissimilar target/decoy pairs. We also show the ranks of the decoys before and after being boosted in the various landscapes. Non-boosted decoy rank is the same as the decoy rank in the no-decoy landscape with the same target noun. SN = Short/Narrow landscape, SW = Short/Wide landscape, TN = Tall/Narrow landscape, and TW = Tall/Wide landscape.

| Word | Frequency |
|---|---|
| dog | 306 |
| cat | 304 |
| car | 176 |
| table | 85 |
| book | 77 |
| love | 76 |
| noun | 71 |
| apple | 68 |
| man | 66 |
| boy | 64 |
| time | 62 |
| lion | 54 |
| house | 52 |
| tree | 50 |
| computer | 39 |
| mother | 38 |
| school | 37 |
| bus | 36 |
| chair | 36 |
| horse | 34 |
| cow | 32 |
| game | 32 |
| art | 31 |
| ball | 31 |
| hand | 31 |
| water | 30 |
| ability | 28 |
| bike | 28 |

| | |
|---|---|
| box | 28 |
| day | 28 |
| pencil | 28 |
| bird | 27 |
| fish | 26 |
| actor | 25 |
| animal | 25 |
| balloon | 25 |
| desk | 25 |
| egg | 25 |
| eye | 25 |
| girl | 25 |
| monkey | 25 |
| king | 24 |
| rat | 24 |
| sun | 24 |
| tiger | 24 |
| spoon | 23 |
| boat | 22 |
| child | 22 |
| father | 22 |
| age | 21 |

Supplementary Table 2 | The 50 most frequently offered words and their frequency in the first round of each game in the experiment.

| Word | Frequency |
|---|---|
| dog | 1711 |
| cat | 1706 |
| car | 1616 |
| table | 1198 |
| book | 1143 |
| noun | 915 |
| love | 903 |
| lion | 902 |
| apple | 848 |
| fish | 843 |
| man | 809 |
| computer | 802 |
| boy | 769 |
| mouse | 768 |
| school | 745 |
| child | 724 |
| girl | 698 |
| bus | 696 |
| tree | 690 |
| bike | 679 |
| bird | 654 |
| pencil | 649 |
| house | 627 |
| water | 621 |
| teacher | 620 |
| tiger | 619 |
| chair | 616 |
| monkey | 597 |

| | |
|---|---|
| eye | 583 |
| day | 582 |
| horse | 562 |
| egg | 548 |
| city | 545 |
| art | 533 |
| king | 532 |
| ball | 530 |
| food | 526 |
| game | 525 |
| hand | 525 |
| flower | 523 |
| mother | 520 |
| moon | 519 |
| hair | 516 |
| animal | 514 |
| train | 511 |
| sun | 500 |
| paper | 499 |
| music | 495 |
| box | 482 |
| father | 478 |

Supplementary Table 3 | The 50 most frequently answered words and their frequency throughout the experiment. Overall, there were 199,436 and 16,082 guesses (included or not included in the list of 20,000 nouns, respectively) by participants in all the games combined, and 6,947 and 4,945 unique words (included or not included in the list of 20,000 nouns, respectively) ever mentioned.

**Supplementary references**


1. Mikolov, T., Chen, K., Corrado, G. & Dean, J. Efficient estimation of word representations in vector space. Preprint at https://doi.org/10.48550/arXiv.1301.3781 (2013).
2. Mikolov, T., Sutskever, I., Chen, K., Corrado, G. & Dean, J. Distributed representations of words and phrases and their compositionality. Preprint at https://doi.org/10.48550/arXiv.1310.4546 (2013).
3. Bird, S., Klein, E. & Loper, E. *Natural Language Processing with Python*. (O'Reilly, 2009).
4. Miller, G. A. WordNet: a lexical database for English. *Commun. ACM* **38**, 39–41 (1995).
5. Arseniev-Koehler, A., Cochran, S. D., Mays, V. M., Chang, K.-W. & Foster, J. G. Integrating topic modeling and word embedding to characterize violent deaths. *Proc. Natl. Acad. Sci. U.S.A.* **119**, e2108801119 (2022).
6. Crouse, D. F. On implementing 2D rectangular assignment algorithms. *IEEE Trans. Aerosp. Electron. Syst.* **52**, 1679–1696 (2016).
7. Virtanen, P. *et al.* SciPy 1.0: fundamental algorithms for scientific computing in Python. *Nat. Methods* **17**, 261–272 (2020).
