# Supplementary material for: Simple autonomous agents can enhance creative semantic discovery by human groups
Source: Nat Commun. 2024 Jun 18;15:5212. doi: 10.1038/s41467-024-49528-y (PMC11189566; doi:10.1038/s41467-024-49528-y)
Supplement: Supplementary file 3 — Reporting Summary [file 41467_2024_49528_MOESM3_ESM.pdf]

nature portfolio

Corresponding author(s): Nicholas Christakis

Last updated by author(s): Jun 4, 2024

Reporting Summary

Nature Portfolio wishes to improve the reproducibility of the work that we publish. This form provides structure for consistency and transparency in reporting. For further information on Nature Portfolio policies, see our Editorial Policies and the Editorial Policy Checklist.

Statistics

For all statistical analyses, confirm that the following items are present in the figure legend, table legend, main text, or Methods section.

n/a	Confirmed	
☐	☒	The exact sample size (n) for each experimental group/condition, given as a discrete number and unit of measurement
☐	☒	A statement on whether measurements were taken from distinct samples or whether the same sample was measured repeatedly
☐	☒	The statistical test(s) used AND whether they are one- or two-sided<br>*Only common tests should be described solely by name; describe more complex techniques in the Methods section.*
☐	☒	A description of all covariates tested
☐	☒	A description of any assumptions or corrections, such as tests of normality and adjustment for multiple comparisons
☐	☒	A full description of the statistical parameters including central tendency (e.g. means) or other basic estimates (e.g. regression coefficient) AND variation (e.g. standard deviation) or associated estimates of uncertainty (e.g. confidence intervals)
☒	☐	For null hypothesis testing, the test statistic (e.g. F, t, r) with confidence intervals, effect sizes, degrees of freedom and P value noted<br>*Give P values as exact values whenever suitable.*
☐	☒	For Bayesian analysis, information on the choice of priors and Markov chain Monte Carlo settings
☐	☒	For hierarchical and complex designs, identification of the appropriate level for tests and full reporting of outcomes
☐	☒	Estimates of effect sizes (e.g. Cohen's d, Pearson's r), indicating how they were calculated

Our web collection on statistics for biologists contains articles on many of the points above.

Software and code

Policy information about availability of computer code

Data collection	Participants were recruited through Amazon Mechanical Turk to participate in the experimental task on a website implemented using Breadboard software (available at http://breadboard.yale.edu).
Data analysis	Analyses were conducted using the RStan v.2.21.8 and its interface brms v.2.19.0 package in R v.4.3.0

For manuscripts utilizing custom algorithms or software that are central to the research but not yet described in published literature, software must be made available to editors and reviewers. We strongly encourage code deposition in a community repository (e.g. GitHub). See the Nature Portfolio guidelines for submitting code & software for further information.

Data

Policy information about availability of data

All manuscripts must include a data availability statement. This statement should provide the following information, where applicable:

- Accession codes, unique identifiers, or web links for publicly available datasets
- A description of any restrictions on data availability
- For clinical datasets or third party data, please ensure that the statement adheres to our policy

The data used in this study have been deposited in the Open Science Framework repository and are available at https://doi.org/10.17605/OSF.IO/CS3R2

Research involving human participants, their data, or biological material

Policy information about studies with human participants or human data. See also policy information about sex, gender (identity/presentation), and sexual orientation and race, ethnicity and racism.

Reporting on sex and gender	Participants' gender was not considered in the study design. Participants' gender was identified based on self-reports asked at the end of the experiment. We did not analyze this variable.
Reporting on race, ethnicity, or other socially relevant groupings	Not applicable.
Population characteristics	Out of the 1,821 participants who completed the post-session questionnaire, 953 identified as male, 848 as female, 12 as non-binary, and 8 preferred not to answer. The mean age of participants was 36.04 (s.d. = 11.22).
Recruitment	Participants were recruited through Amazon Mechanical Turk to participate in the experiment.
Ethics oversight	This study was approved by the Yale University Committee on the Use of Human Subjects. All the ethical regulations were met in conducting the current study. Prior to beginning the task, all participants gave informed consent as approved by the Yale University Committee on the Use of Human Subjects.

Note that full information on the approval of the study protocol must also be provided in the manuscript.

Field-specific reporting

Please select the one below that is the best fit for your research. If you are not sure, read the appropriate sections before making your selection.

☐ Life sciences ☒ Behavioural & social sciences ☐ Ecological, evolutionary & environmental sciences

For a reference copy of the document with all sections, see nature.com/documents/nr-reporting-summary-flat.pdf

Behavioural & social sciences study design

All studies must disclose on these points even when the disclosure is negative.

Study description	Quantitative experimental.
Research sample	Participants were recruited through Amazon Mechanical Turk (MTurk) to participate in the experiment. Out of the 1,821 participants who completed the post-session questionnaire, 953 identified as male, 848 as female, 12 as non-binary, and 8 preferred not to answer. The mean age of participants was 36.04 (s.d. = 11.22).
Sampling strategy	We used a sample recruited through Amazon Mechanical Turk. The sample size was based on past work that has a similar experimental protocol (Shirado & Christakis, 2017).
Data collection	Participants used their personal computers and keyboards to record their responses. The researcher was not present besides the participants during data collection.
Timing	Start: January 8, 2023. End: April 29, 2023.
Data exclusions	No data was excluded for the analyses. We described exclusion criteria in the preregistration. The preregistration is available at https://doi.org/10.17605/OSF.IO/X8GWS
Non-participation	Out of the 1,875 participants, 54 participants did not respond to the post-session questionnaire probably due to issues in the Internet connection.
Randomization	Participants were allocated to each experimental condition according to the fractional factorial design as described in the preregistered protocol (available at https://doi.org/10.17605/OSF.IO/X8GWS), and their position within the networked groups was random.

Reporting for specific materials, systems and methods

We require information from authors about some types of materials, experimental systems and methods used in many studies. Here, indicate whether each material, system or method listed is relevant to your study. If you are not sure if a list item applies to your research, read the appropriate section before selecting a response.

Materials & experimental systems

n/a	Involved in the study
☒	☐ Antibodies
☒	☐ Eukaryotic cell lines
☒	☐ Palaeontology and archaeology
☒	☐ Animals and other organisms
☒	☐ Clinical data
☒	☐ Dual use research of concern
☒	☐ Plants

Methods

n/a	Involved in the study
☒	☐ ChIP-seq
☒	☐ Flow cytometry
☒	☐ MRI-based neuroimaging

Plants

Seed stocks	*Report on the source of all seed stocks or other plant material used. If applicable, state the seed stock centre and catalogue number. If plant specimens were collected from the field, describe the collection location, date and sampling procedures.*
Novel plant genotypes	*Describe the methods by which all novel plant genotypes were produced. This includes those generated by transgenic approaches, gene editing, chemical/radiation-based mutagenesis and hybridization. For transgenic lines, describe the transformation method, the number of independent lines analyzed and the generation upon which experiments were performed. For gene-edited lines, describe the editor used, the endogenous sequence targeted for editing, the targeting guide RNA sequence (if applicable) and how the editor was applied.*
Authentication	*Describe any authentication procedures for each seed stock used or novel genotype generated. Describe any experiments used to assess the effect of a mutation and, where applicable, how potential secondary effects (e.g. second site T-DNA insertions, mosiacism, off-target gene editing) were examined.*
